# Olov Oscarsson’s Description of Afferent Pathways to the Cerebellum: Excellent Physiology, Base for Anatomy, and Road Toward Understanding Function

**DOI:** 10.1007/s12311-023-01516-6

**Published:** 2023-01-24

**Authors:** Tom J. H. Ruigrok

**Affiliations:** Department of Neuroscience, Erasmus University Medical Center, Rotterdam, The Netherlands

**Keywords:** Spino-olivocerebellar, Climbing fibers, Inferior olive, Mossy fibers, Cerebellar afferents

## Abstract

Olov Oscarsson’s review on the functional organization of spinocerebellar paths is a prime demonstration of the great skills and huge knowledge base of the electrophysiologists of his era working on communication systems in the brain. Oscarsson describes and characterizes in detail no less than ten different communication lines between the spinal cord and the cerebellum. As such, his work proved to be a highly fertile basis for ongoing physiological and anatomical research. However, even after 50 years of continuing cerebellar research, many questions are still open and even care must be taken that the differentiation in spinocerebellar paths, so carefully demonstrated by Oscarsson, is not lost in present-day research.

This issue’s *Cerebellar Classic* is a review by Olov Oscarsson highlighting the beautiful work he and his group performed on cerebellar afferent systems in Lund, Sweden, in the 1960s and 1970s [1]. By combining high-standard electrophysiological techniques with clever experimental set-ups, they succeeded in accumulating a wealth of data on the great variety of pathways that supply the cerebellum with information. In his review, no less than ten distinct spinocerebellar pathways are discussed. Each of these pathways could be differentiated and characterized by the location of their ascending fibers (tracts), their specific transmission latencies and activation by peripheral and/or central afferents, and, at least to some extent, their different cerebellar terminal regions.

In describing these paths, this review clearly shows the incredible workmanship, dedication, and physiological knowledge of not just Oscarsson’s group but also of many scientists of that era who worked on communication between the peripheral nervous system, the spinal cord, and the brain. One wonders how much of that vast knowledge is presently still operative by today’s scientists carrying out behavioral studies with electrophysiological techniques. Of course, one should take into account that in those days the cat was often the experimental animal of choice, whereas later, cat research was mostly abandoned in favor of experiments with rodents, such as rats and, in particular, mice. It is obvious that despite obvious advantages and numerous technological advances, the size of the mouse also has its limitations. Nevertheless, available knowledge suggests that spinocerebellar systems in cat and mouse are, at least to some extent, similarly organized [2].

One of the highlights of Oscarsson’s review is that, using physiological techniques, it was possible to demonstrate the termination patterns of the various mossy and climbing fiber pathways. As such, it was one of the first demonstrations that the strict longitudinal, stripe-like pattern of climbing fiber projections, as shown by selective activation of the various spino-olivocerebellar pathways (SOCPs), was quite different from the widely, divergent, terminal reach of the spinocerebellar mossy fiber systems. However, despite a half century of further research, many questions concerning their termination patterns are still open. For instance, it is still a point of study and debate to what extent the ascending spinocerebellar and descending cortico-pontocerebellar pathways are separated, locally interact, or are really integrated, not only in the granule cell layer [3] but also by the parallel fiber system into the molecular layer. In addition, although the divergent nature of the mossy fiber system still clearly contrasts that of the climbing fiber system, it has become evident that a topographical relation is also present [4].

Interesting ideas, discussed in the review, that still have not been either discarded or verified, are the potentially labeled information lines to Purkinje cell (PC) dendrites. Specific information supplied by, e.g., the dorsal spinocerebellar tract seems to terminate in deeper parts of the granular layer. These granule cells give rise to parallel fibers in the deeper parts of the molecular layer, potentially terminating on proximal dendrites of the PCs. On the other hand, spinocerebellar fibers carrying more a-specific information seem to preferentially terminate upon more superficial granule cells. This idea, based on observations by Szentágothai [5], was also mentioned as potentially highly relevant in a recent review of granule cell patterning [6]. However, to what extent this really can be considered an important functional difference has not yet been established [7].

Perhaps the most striking and well investigated feature of Oscarsson’s work has been the observation that the physiologically defined SOCPs can be related to the anatomical organization of the olivo-cortico-nuclear connectivity [8]. Indeed, due to the similarity of PC activation patterns induced by stimulation of the various SOCPs and the stripe-like organization of olivary climbing fibers on parasagittally organized bands of PCs that target the various parts of the cerebellar nuclei, it was decided to refer to the physiologically recognized bands with similar, but lowercase letters as Voogd’s zones that were indicated with capitals [9, 10]. Subsequent studies, with detailed mapping of SOCPs in rat and olivo-cortico-nuclear mapping in rat and mouse, compared their organization with the pattern of intrinsic chemical markers in PCs and proposed that the physiological, anatomical, and chemical patterns are all representative of a single map [11].

Here, a further mention of Oscarsson’s observation on SOCPs should be made, as the five distinguished paths not only take a different route to the inferior olive but are also activated by different afferent sources and terminate in different longitudinal zones of the cerebellar anterior lobe and paramedian lobule and pyramis. This suggests that olivary neurons are functionally diverse, a fact that would reflect different functional roles of the related cerebellar zones. Indeed, it was already postulated by Oscarsson that these cortical zones, which, as mentioned above, are interconnected with specific parts of the inferior olive as well as with parts of the cerebellar nuclei, gave rise to the concept of parallelly organized olivo-corticonuclear modules [12], each having a specific but different function to fulfill for motor control. However, despite several attempts, it is still not clear, firstly, to what extent the modules represent individual and functionally diverse units, and secondly, what these different modular functions might be [13].

As a final comment, a word of caution should be added. In Oscarsson’s review, at least five paths, originating in the spinal cord (and dorsal column nuclei) are distinguished that terminate as mossy fibers in the cerebellum. It is noteworthy that the term “spinocerebellar” was mentioned in 12 papers in the year this review was published, whereas in 2021 it occurred in the title or keywords of at least 469 articles (source Pubmed). However, in most cases the term was used in combination with “ataxia.” The phrase “spinocerebellar tract” only appeared in 4 publications of that year and in only one of these, the distinction between the various spinocerebellar tracts was made with references to the work of Oscarsson and colleagues [14]. This suggests that some concern may be justified that the functional and structural identity of the diversification of the spinocerebellar tracts tends to be underrated. In the recent paper by Pop et al. [14], it was possible to demonstrate that the spinocerebellar pathway originating from the column of Clarke consisted of neurons with a *Neurog1*-lineage, whereas the indirect spino-reticulocerebellar pathway and spino-olivocerebellar paths are all derived from *Atoh1*-lineage neurons. Yet, no further differentiation of the various pathways seemed possible or was mentioned. Indeed, although the development of the different spinocerebellar tracts has been shown to be controlled by *Hox*-dependent genetic programs [15] and several molecular differences have been noted [16], presently, as far as we know, no definite genetic differentiation of the various origins and pathways has been demonstrated. Yet, further research into the genetic diversification of the various spinocerebellar pathways seems to be the way in order to come to grasp with cerebellar processing of afferent information.

In conclusion, Oscarsson’s work has been, and still is, an inspiring basis for past, present-day, and future scientific work. Anyone working on motor control and learning by the cerebellum will be inspired and motivated by the truly amazing work of the many excellent electrophysiological scientists of the last century, of which Olov Oscarsson was a prime representative.
